# Biomarker discovery using intact, molecularly active postmortem whole human Alzheimer's disease brains

**DOI:** 10.1002/alz70861_108055

**Published:** 2025-12-23

**Authors:** Paul D Wes, Josip Butkovic, Karina Wong, Brian Hinger, Randall Buck, Chunmin Ge, Congwei Wang, Brad Parry, Zvonimir Vrselja

**Affiliations:** ^1^ Bexorg, New Haven, CT USA

## Abstract

**Background:**

The availability of preclinical models that accurately predict efficacy in patients remains a major obstacle in advancing therapeutics for Alzheimer’s disease (AD). To address this gap, we have established an ex vivo culture system to maintain metabolically and cellularly active whole intact postmortem brains, including AD brains.

**Methods:**

Brains were isolated from postmortem human donors, following the highest ethical standards for tissue donation, as well as from pigs. A novel perfusion system was established to physiologically maintain the brains for 24 hours without reinitiating network activity associated with consciousness.

**Results:**

Optimization of the perfusion system enabled us to achieve sustained cerebral circulation, maintenance of blood‐brain barrier, and recovery of cellular and molecular functions after prolonged global anoxia in human brain. We demonstrated the application of neuroanatomical and viral vectors in major brain regions for investigation of long‐range neuronal connectivity. Furthermore, we provide evidence of functional gene delivery to and across brain vasculature using systemically administered clinical AAV vectors. To demonstrate the utility of the platform for small molecule drug discovery, benchmark tool molecules were systemically delivered, and compound levels were measured in brain tissue and perfusate in real‐time using Raman spectroscopy. Moreover, metabolomic and proteomic response to drug was measured in the venous output of the brain, providing translational biomarkers for treatment response.

**Conclusions:**

Our data show that our perfusion‐based postmortem brain model can recapitulate the complexity of the brain at the cellular and systems level. This paves the way for conducting preclinical drug discovery in postmortem human disease brain, including demonstration of drug exposure at the site of action, target engagement, and functional impacts on disease‐relevant endpoints, as well as optimization of brain delivery technologies. Utilizing human disease brains as a preclinical model promises to substantially increase the probability of success in developing new therapies for AD.

